# Does Long-Term Training in a Water Immersion Environment Change Interoception?

**DOI:** 10.3390/ijerph181910259

**Published:** 2021-09-29

**Authors:** Yasuhiro Baba, Daisuke Sato, Naofumi Otsuru, Koyuki Ikarashi, Tomomi Fujimoto, Koya Yamashiro

**Affiliations:** 1 Department of Health and Sports, Niigata University of Health and Welfare, 1398 Shimamicho, Kita-Ku, Niigata 950-3198, Japan; daisuke@nuhw.ac.jp (D.S.); fujimoto@nuhw.ac.jp (T.F.); yamashiro@nuhw.ac.jp (K.Y.); 2 Major in Health and Welfare, Graduate School of Niigata University of Health and Welfare, 1398 Shimamicho, Kita-Ku, Niigata 950-3198, Japan; hwd21002@nuhw.ac.jp; 3 Institute for Human Movement and Medical Sciences, Niigata University of Health and Welfare, 1398 Shimamicho, Kita-Ku, Niigata 950-3198, Japan; otsuru@nuhw.ac.jp; 4 Department of Physical Therapy, Niigata University of Health and Welfare, 1398 Shimamicho, Kita-Ku, Niigata 950-3198, Japan

**Keywords:** interoceptive accuracy, water immersion, heart rate, heartbeat counting task, swimmer, baseball player, interoception

## Abstract

The aim of this study was to investigate individual interoception by comparing the responses of swimmers and baseball players when exposed to specific water environments, depending on training content and environment. Forty-eight healthy male university students were evaluated for their interoceptive response (accuracy, sensibility, and awareness) and heart rate following 25 min of water immersion (WI) at 35 °C. We assessed three conditions: pre-WI, during WI, and post-WI. The results indicated that interoceptive accuracy (IAcc) did not differ between groups because both swimming and baseball do not require emotional expression, as opposed to an activity such as dance. The heart rate was significantly decreased at post-WI compared to that at pre-WI. The IAcc of post-WI presented as higher than that of pre-WI. In addition, there was a significant negative correlation between the ratio of IAcc and that of HR among subjects. Moreover, the attention regulation subscale of the MAIA changed in the WI environment and the ratio of IAcc was negatively correlated with that of the not-distracting subscale of the MAIA. These results suggested that interoception did not differ among the athletes who had long-term training, which enabled them to acquire multi-modal sensorimotor integration, compared to that of non-athlete control participants. We conclude that interoception did not differ among athletes who had long-term training compared to that of non-athlete control participants.

## 1. Introduction

Interoception is defined as the perceptual process that gives us a sense of the physical body from within [1,2], a topic on which sensory research began 100 years ago [3]. Physiological mechanisms acting as interoceptive stimuli comprise proprioceptive and visceroceptive processes, such as heart rate, respiration, blood pressure, and gastrointestinal or genitourinary activity [4]. Bodily sensations arising from homeostatic processes in the body (e.g., heart rate changes, temperature, hunger, arousal, touch, itch, and gut motility) are crucially related to the conscious experience of affect [5,6,7,8] and to the creation of selfhood [2]. Thus, interoception is considered a key perceptual system for consciousness and self-awareness [1,9,10,11].

Three main dimensions of interoception are assessed [12]. The first is interoceptive accuracy (IAcc), which is the objective accuracy of perceiving bodily signals such as heart rate (HR) and gastric activity [13,14,15,16]. IAcc is usually assessed using the heartbeat counting task, in which participants are instructed to silently count their heartbeats for several time intervals and only focus on their internal bodily signals at the time [13,17]. Second, interoceptive sensibility (IS) is evaluated by a self-rated tendency to focus on internal bodily signals as reported in questionnaires (e.g., Multidimensional Assessment of Interoceptive Awareness (MAIA) and the 20-item Toronto Alexithymia Scale (TAS-20): a classic measure of alexithymia, which may be related to interoception) [18,19]. The last dimension is interoceptive awareness (metacognitive insight of IAcc) evaluated by a visual analog scale (VAS) [20,21] or a receiver operating characteristic (ROC) curve [12]. As these three dimensions have no common correlations [12], they need to be measured separately.

Previous studies have reported that the process of interoception includes physiological and psychological aspects, sympathovagal balance modulation [22], cardiorespiratory activity [23], respiratory activity [24], body ownership [25], self-objectification [26,27], and emotion regulation [17,28,29,30]. Interestingly, several studies have reported that physical activity and/or experience of sports are related to interoception [24,31,32,33,34,35]. Several studies have indicated higher IAcc in athletes with long-term training than in controls, and explained that long-term training-induced HR changes seem to be a factor that affects interoception [24,32,35]. For instance, Christensen et al. [32] reported that professional dancers showed higher IAcc scores compared to control participants and considered lower HR as an explanation for this result. On the other hand, a measurement of IAcc in the heartbeat counting task showed no significant difference between a group of 17 long-distance runners and a team of 13 sprinters, despite differences in HR between these groups [36]. Both groups included males and females; therefore, the role of HR in IAcc is still under consideration. In addition, it is unclear whether the intra-individual alteration of HR affects IAcc scores. Another possible explanation for higher IAcc in athletes is the repetitive multi-modal sensorimotor integration that occurs throughout long-term training. Previous studies have reported that long-term training of movement or exercise that requires multi-modal sensorimotor integration (e.g., visual, auditory, and somatosensory), such as dance and the performance of music, could improve IAcc scores [32,37]. Considering that the required sensorimotor integration differs depending on participation in sports, characteristics of those experienced in sports may be reflected in IAcc.

Moreover, it has been reported that the brain activity of athletes changes in a manner dependent on training environment. It has been indicated that swimmers, who usually process a large amount of somatosensory information such as touch, pressure, temperature, and cold during training in an underwater environment, can maintain the same sensory–motor functions in an underwater environment as on land [38]. In contrast, in non-swimmers, water-induced somatosensory input causes unnecessary brain activity, resulting in a decrease in sensory–motor functions in an underwater environment [38]. Thus, responses in a water immersion (WI) environment may differ between swimmers and non-swimmers who have trained in the water.

We present two hypotheses. First, contextual long-term training in a water environment will change swimmers’ IAcc scores, which might be higher than that of dry-land athletes. The second hypothesis is that athletes who continue to train in a special environment will be able to maintain their IAcc scores in that environment. To test these two hypotheses, this study compared the interoceptive responses of athletes of two types of sports (swimming and baseball) and non-athletes (control). The aim of this study was to investigate whether individual interoception and its response differ when exposed to specific environments depending on the training content and environment, by comparing swimmers to baseball players.

## 2. Methods

### 2.1. Participants

Seventeen healthy swimming athletes, fifteen baseball players, and sixteen controls without experience in sports participated in the present study (see Table S1 for details on participants’ characteristics). All participants were males who attended the same university in Japan. Furthermore, all swimmers belonged to the same swimming team at this university and had experience competing at national or intercollege swim meets. Fédération Internationale De Natation (FINA) points were calculated from their own fastest records to evaluate swimmers’ performance levels (Swimming points of FINA. Available online: http://www.fina.org/content/fina-points (accessed on 16 August 2021)). The mean FINA points of the swimmers were 730.4 ± 13.5. The non-swimmers were university students with no training in swimming and baseball. The sample size was determined based on previous work and calculated using GPower 3.1. [39], which indicates that groups of 15 swimmers, 15 other athletes, and 15 controls would be sufficient to detect a similar effect with a power of 85%. The effect size of *f* = 0.4. To maintain reliability, we recruited 17 swimmers, 15 baseball players, and 16 control subjects. The three groups of participants were matched in age. Swimmers, baseball players, and control groups of mean ages were 18.5 ± 0.6, 19.9 ± 0.4, and 19.3 ± 0.3, respectively. Swimmers had been training for 14.1 ± 3.0 years, and baseball players, for 11.5 ± 2.2 years. The mean number of years of training experience in the control groups was 2.3 ± 2.4 years, which was at least one year after stopping continuous training. The mean Body Mass Index (BMI) of the swimmers, baseball players, and controls was 23.1 ± 1.3, 24.6 ± 1.3, and 21.2 ± 2.4, respectively. The participants provided informed consent. This study was approved by the ethics committee of the Niigata University of Health and Welfare, Japan. All experiments conformed to the Declaration of Helsinki and were approved by the ethics committee of the Niigata University of Health and Welfare, Japan (approval number 18494).

### 2.2. Experimental Protocol

**Figure 1:**
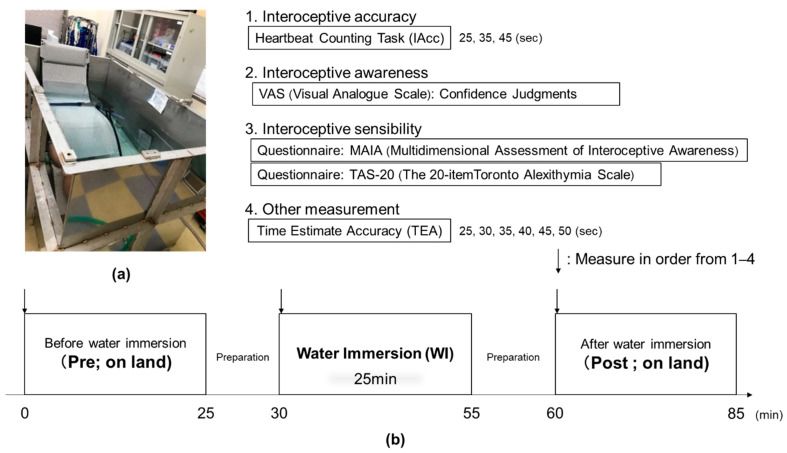
Experimental protocol in this study. (**a**) Reclining armchair in water (**b**) The above shows the interoceptive measurements (1–4) and bottom shows Protocol.

Experiments were performed in the afternoon, and none of the swimmers had swum beforehand. Participants wore only swimwear and were seated on comfortable reclining armchairs with mounted headrests (Figure 1a). After preparation for IAcc, trials were performed before, during, and after 25 min of WI, as shown in Figure 1b. Each trial consisted of tests for IAcc, interoceptive sensibility, interoceptive awareness, and time estimation accuracy. The water and ambient temperatures were maintained at 35 °C and 28 °C, respectively, because the metabolic response is less sensitive at these temperatures. A water temperature of 35 °C is considered neutral water temperature. In addition, the thermosensory stimulation from the skin due to temperature was kept to a minimum during the experiment. Water was poured up to the axillary level of each participant during WI. 

Interoceptive responses were measured before, during, and after 25 mins of water immersion.

### 2.3. Interoceptive Accuracy Data

First, participants performed a heartbeat counting task. HR was monitored with an electrocardiographic HR sensor (DL-310, S&ME Inc., Tokyo, Japan) attached to the participant; sampling was at 1 kHz, which recorded the derived electrical signal onto a second PC using Lab Chart 8 software (AD Instruments). Participants’ HRs were recorded throughout the experiment by an AD Instruments Power Lab System (Power Lab. 8/35, AD Instruments, Oxfordshire, UK), including an analog output (DL-720, S&ME Inc., Tokyo, Japan). Self-adhesive electrodes were attached to participants’ abdomens, and a ground electrode was attached to their clavicles. Lab Chart 8 (v.8.1.13, 1994–2016, AD Instruments, Oxfordshire, UK) was used to record and analyze the ECG signal from which the HR was derived. A trigger was sent from E-Prime to the ECG trace to demarcate the onset and offset of each trial. Heartbeat perception was measured using the mental tracking method [40], which has been widely used to assess interoceptive awareness, has good test–retest reliability, and correlates highly with other heartbeat detection tasks [41]. Participants were instructed to silently count their own heartbeats on an audiovisual start cue until they received an audiovisual stop cue. After one brief training session (15 s), the experiment began. It consisted of three different time intervals of 45 s, 35 s, and 25 s, presented in a random order across participants. Participants were asked to type the number of heartbeats counted at the end of each interval. Throughout, participants were not permitted to take their pulse, and no feedback on the length of the counting phases or the quality of their performances was given. IAcc scores were calculated as:

[IAcc = 1 − 1/nΣ |(recorded heartbeats − counted heartbeats)|/recorded heartbeats] [13,42].

Higher scores indicated a higher IAcc. A higher score indicates a smaller error between the count and the actual measurement: 1 for perfect and no difference. The lower the score, the closer it is to 0. HR is known to correlate with IAcc, and was recorded during the heartbeat perception task [5,43,44].

### 2.4. Interoceptive Sensibility Data

We used two self-report questionnaires. The questionnaires were not modified to measure the latest states (pre, MI, and post); participants were instructed to respond to the state at that moment. Because the Japanese version of the questionnaire was utilized, items with low values of Cronbach′s α were excluded from the analysis.

#### 2.4.1. Multidimensional Assessment of Interoceptive Awareness (MAIA) Questionnaire

The MAIA was based on a conceptual delineation of multiple interoceptive perception processes and was represented by a 32-item self-reporting questionnaire with 8 scales [19]. The scales were: (1) noticing (perception of unpleasant, pleasant, and neutral body sensations), (2) not-distracting (tendency not to ignore or not be distracted by sensations of pain or discomfort), (3) not-worrying (tendency not to worry or experience emotional distress about sensations of discomfort or pain), (4) attention regulation (ability to maintain and control attention to bodily sensations), (5) emotional awareness (awareness of the relationship between bodily sensations and states of emotion), (6) self-regulation (ability to modulate distress by attention to bodily sensations), (7) body listening (actively listening to the body for some insight), and (8) trusting (experience that one’s body is safe and trustworthy). The items were answered on a six-point Likert scale (0–5), with higher scores indicating greater interoceptive body awareness. The MAIA was administered prior to the start of each heartbeat counting experiment. We used the Japanese version of the MAIA [45]. The Cronbach’s α values of all the MAIA subscales were high, ranging from 0.72 (not-distracting) to 0.87 (attention regulation), except for one (not-worrying). Similarly, an intra-class correlation coefficient (ICC) of the Japanese version of the MAIA was 0.74–0.87. However, the not-worrying subscale included in the original structure had poor (α  =  0.32) and slightly less test–retest reliability (ICC  =  0.68).

#### 2.4.2. Twenty-Item Toronto Alexithymia Scale (TAS-20) Questionnaire

The TAS-20 [18] was used to measure alexithymia and has three subscales: Difficulty Identifying Feelings subscale (DIF), measuring difficulties in identifying emotions (five items); Difficulty Describing Feeling subscale (DDF), measuring difficulties in identifying emotions (seven items); and Externally Oriented Thinking subscale (EOT), which measures the tendency to focus attention externally (eight items). The items are rated on a scale from 1 to 5, where 1 is equivalent to strongly disagree and 5 is equivalent to strongly agree. Five items were negatively keyed and back-transformed when calculating sums. The total alexithymia score is the sum of responses to all 20 items, with possible scores ranging from 20 to 100, with higher scores indicating higher degrees of alexithymia. Cut-offs in TAS-20: scores equal to or less than 51 = non-alexithymia, 52–60 = moderate alexithymia, and equal to or greater than 61 = clinical alexithymia. TAS-20 can also be used as a continuous variable when studying the degree and tendency of alexithymia in various groups. The subscales measuring various aspects of alexithymia can range from 5 to 25 for DID, 7 to 35 for DDF, and 8 to 40 for the EOT subscale. Higher scores indicate higher degrees of the corresponding aspects of alexithymia. Subscales were used as continuous variables [46]. The TAS-20 [18] includes 20 items that ask about a person’s difficulty in identifying and describing their own feelings as well as their tendency for externally focused thinking. Moreover, the application of TAS-20 in highly diverse cultures supports the use of the scale in cross-cultural research [47]. We used the Japanese version of the TAS-20 questionnaire, which is reliable for use in the Japanese population [48]. The Cronbach’s α values of the TAS-20 subscales of DIF and DDF were higher than 0.6, except for one (EOT).

### 2.5. Interoceptive Awareness Data (Confidence Judgments)

At the end of each IAcc (heartbeat counting) task, the participants immediately rated their confidence in their response accuracy. Participants were scored on a vertical line by a pencil mark on a continuous visual analogue scale (VAS) that was on 100 mm long printed paper. The VAS question was “Can you accurately report when your heart is beating?” with anchors of not at all confident (0) to very confident (100) in Japanese.

### 2.6. Time Estimation Accuracy Data

The heartbeat counting task was contaminated by time estimation and knowledge of HR under the original task instructions [49]. We measured the perceptions of time [50] to discern the relationships between the perceptions of IAcc and time estimation accuracy (TEA). Participants counted, in seconds, for six time intervals (25 s, 30 s, 35 s, 40 s, 45 s, and 50 s). TEA was calculated as:

[TEA = 1 − 1/nΣ |(actual time − answered time)|/actual time].

### 2.7. Statistical Analysis

Parametric data (distribution confirmed by the Shapiro–Wilk test) were entered into a two-factor mixed design ANOVA where “group” (swimmer, baseball, and control) was the between-subject’s factor and “environment” (pre, WI, and post) was the within-subject’s factor. Following the mixed design ANOVAs, the Greenhouse–Geisser correction was used to correct for non-sphericity if necessary, and Bonferroni’s post hoc tests were used for pairwise comparisons when applicable. Additionally, the relationships between the change in IAcc and other parameters were analyzed using Pearson’s correlation coefficient. Statistical significance was set at *p* < 0.05. Data were analyzed using the Statistical Software Package (IBM SPSS Version 27, Armonk, NY, USA). All data are expressed as the mean ± SD.

## 3. Results

### 3.1. Interoceptive Accuracy and Interoceptive Awareness (Confidence Judgments)

**Figure 2:**
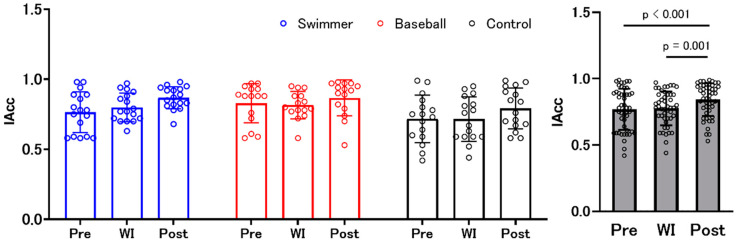
Changes in interoceptive accuracy (IAcc) scores in swimmers, baseball players, and control. Blue, red, and black bars indicate the swimmer, baseball, and control groups, respectively. There was no significant difference among the groups. Gray bars represent all participants with individual data (open circles). IAcc scores at post-water immersion (WI) were higher than those at pre-WI and during WI.

Results from the two-factor mixed design ANOVA revealed that there was a significant main effect of “environment” (F (1.760, 3.519) = 9.689, *p* < 0.001, ηp^2^ = 0.177), but not “group” × “environment” interaction effect, and a main effect of “group.” The post hoc test within “environment” showed significantly higher IAcc scores after WI (post) compared to that before and during WI (pre and WI) (Figure 2). The changes in IAcc scores in each type of task are presented in the Supplementary Materials (Figure S1).

**Figure 3:**
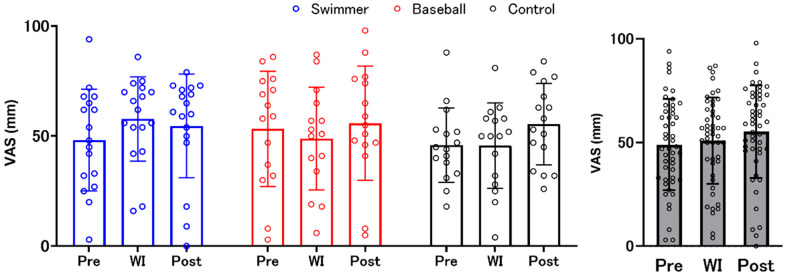
The confidence level for their answers. Blue, red, and black bars indicate the swimmer, baseball, and control groups, respectively. There was no significant difference among the groups. Gray bars represent all participants with individual data (open circles). VAS: visual analogue scale. WI: water immersion.

The confidence level for their answers was evaluated using the VAS. Results from the two-factor mixed design ANOVA revealed no significant interaction (F (4, 90) = 1.377, *p* = 0.248, ηp^2^ = 0.058) or main effects of “environment” (F (2, 90) = 2.267, *p* = 0.110, ηp^2^ = 0.048) and “group” (F (2, 45) = 0.277, *p* = 0.759, ηp^2^ = 0.12) (Figure 3).

### 3.2. MAIA (Interoceptive Sensibility)

Results from the two-factor mixed design ANOVA revealed significant main effects of “group” in attention regulation, self-regulation, body listening, and trusting, with higher scores in the baseball group compared to the control group, followed by a post hoc test (Table 1). This analysis showed that the significant main effect of “environment” was shown only in attention regulation, but not in other domains, and no interaction effect in all domains.

### 3.3. TAS-20

Results from the two-factor mixed design ANOVA revealed a significant main interaction between “group” and “environment”, only in EOT, with a higher score at post compared to at WI in the swimmer group (*p* = 0.006), but not in other domains (Table 1). There were significant main effects of “environment” in the total score and DIF but not in other domains, and no main effect of “group” in all domains.

### 3.4. Time Estimation Accuracy

**Figure 4:**
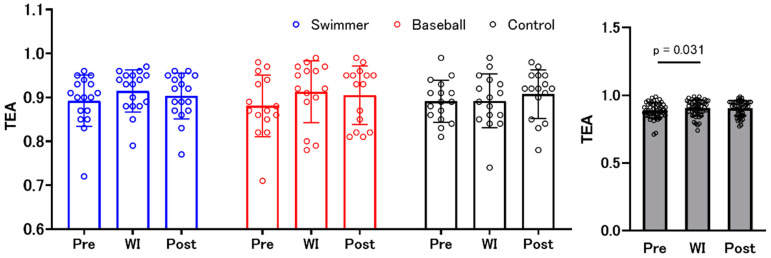
Changes in time estimation accuracy. Blue, red, and black bars indicate the swimmer, baseball, and control groups, respectively. There was no significant difference among the groups. Gray bars represent all participants with individual data (open circles). Time estimation accuracy (TEA) was significantly higher during water immersion (WI) than before WI (pre-WI).

Results from the two-factor mixed design ANOVA revealed that there was a significant main effect of “environment” (F (2, 90) = 3.699, *p* = 0.029, ηp^2^ = 0.076), but not a “group” × “environment” interaction effect and main effect of “group”. The post hoc test within “environment” showed significantly higher TEA at WI compared to that before WI (pre) (Figure 4). The changes in TEA in each type of task are presented in the Supplementary Materials (Figure S2).

### 3.5. Heart Rate

**Figure 5:**
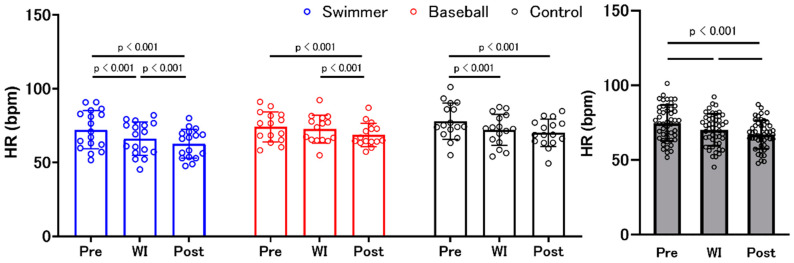
Changes in heart rate. Blue, red, and black bars indicate the swimmer, baseball, and control groups, respectively. Gray bars represent all participants with individual data (open circles). Heart rate (HR) significantly decreased at post compared to that at pre in all groups, during water immersion (WI) compared to that at pre in swimmer and control groups, and at post compared to that during WI in swimmer and baseball groups.

Results from the two-factor mixed design ANOVA revealed that there was a significant “group” × “environment” interaction effect (F (2, 90) = 3.508, *p* = 0.010, ηp^2^ = 0.135) and main effect of “environment” (F (1.691, 3.381) = 80.759, *p* < 0.001, ηp^2^ = 0.642), but not a main effect of “group.” The post hoc test showed a significant HR decrease at post compared to that at pre (Figure 5).

### 3.6. Correlation between the Change in IAcc Scores and Other Parameters

**Figure 6:**
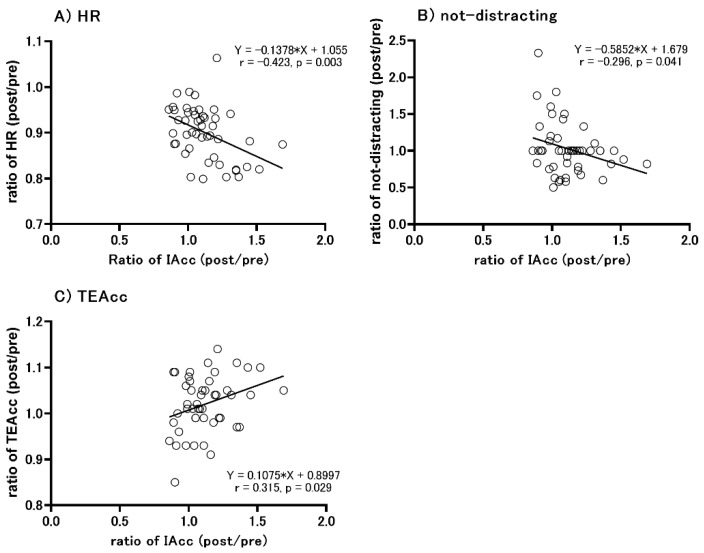
Correlation between the change in the ratio of interoceptive accuracy (IAcc) and other parameters. The x-axis is the difference in the ratio of IAcc (post-water immersion (WI)/pre-WI). The y-axis is the difference in HR (**A**), not-distracting in the Multidimensional Assessment of Interoceptive Awareness (MAIA) (**B**), and time estimation accuracy (TEAcc) (**C**). Not-distracting indicates the tendency not to ignore or distract oneself from sensations of pain or discomfort.

Results from the Pearson correlation analysis revealed that the change in IAcc pre-and post-intervention was significantly correlated with the changes in HR (r = −0.423, *p* = 0.003, Figure 6A), but not with MAIA (r = −0.296, *p* = 0.041, Figure 6B) and the ratio of TEAcc (r = 0.315, *p* = 0.029, Figure 6C), and not with other parameters.

## 4. Discussion

The present study examined whether the contents and environments of long-term training affect interoception by comparing swimmers, baseball players, and control participants. The main findings of this study are as follows: (1) long-term training of swimming and baseball did not affect interoception; (2) the intervention of WI increased interoception not only in swimmers but also in baseball players and control participants, contrary to our hypothesis that only the swimmers could maintain interoception in water as well as on land.

The results of the present study revealed that there was no significant difference in the IAcc between athletes who continued training for a long time and the control group, contrary to our hypothesis. One explanation for the lack of difference among these groups is that HR did not differ among them. A previous study that compared IAcc between dancers and control participants reported that IAcc scores were higher in dancers because of their lower HR at rest [32]. Another study [51] indicated that there was a significant negative correlation between IAcc and resting HR. In the present study, there was no difference in resting HR among the three groups and a significant negative correlation between the ratio of HR and the ratio of IAcc within subjects, which would have influenced the results of the IAcc scores (Figure 6). Second, the characteristics of experience in sport in the athlete groups may also be involved in the lack of difference in IAcc. Previous studies have reported that long-term training of movement or exercise that requires multi-modal sensorimotor integration (e.g., visual, auditory, and somatosensory), such as dance and the performance of music, could improve IAcc [32,37]. On the other hand, swimming and baseball, which the present participants have experienced, have characteristics that athletes experience mainly based on visual and somatosensory information [38,52,53]. Dancing and/or playing music involves more auditory stimuli than swimming does. In addition, long-term experiences in which emotions are expressed through dancing and playing music may enhance IAcc because emotions such as empathy and sympathy are also strongly associated with IAcc [54]. Therefore, the present results of no differences between athletes and control subjects may be because their sport movement is based on visual and somatosensory information and does not require emotional expression. The other explanation is that all the present participants were Japanese, as opposed to previous participants. Ma-Kellams [55] examined individual differences of culture, language, and race on interoception, and illustrated that East Asians were more sensitively aware of the state of the body than Westerners were. Additionally, awareness of the state of the body is widely used as a methodology to improve interoception [29]. Therefore, the present study recruited only Japanese individuals (East Asian subjects), who might influence a different outcome than previous studies.

Interestingly, the present results indicate no difference of interoception between individual (swimming) and team (baseball) sports. To our knowledge, this is the first study to explore the difference between these sports. Several studies reported that adventure racers [24] or warfighters [35], who continue to train hard in groups, showed higher interoception. However, these studies have not compared their interoceptions with those of athletes of individual sports. Additionally, higher interoceptions were explained by athletes’ experiences of continuous activities in extreme conditions. Although we cannot conclude that there is no difference in interoception between individual and team sports due to such limited existing research, it seems that there is no difference between swimmers and baseball players.

The present study revealed that the intervention of WI increased IAcc scores in all groups, contrary to our hypothesis that only swimmers but not others could maintain IAcc scores in water and on land. One possible explanation for the improvement of IAcc after WI (post) could be a decrease in HR. As described above, cross-sectional correlated analysis has shown that inter-individual differences in IAcc were related to resting HR [32,51]. However, it remains unclear whether this correlation between IAcc scores and resting HR holds for intraindividual differences.

The present results reveal that intra-individual changes in HR at rest significantly correlated with changes in IAcc, which extends the findings of previous studies. However, we need to consider the reason that IAcc scores did not change during WI despite the significant decrease in HR. This would explain why somatosensory processing induced by WI affects the IAcc during WI. During WI, we receive several continuous somatosensory inputs from a wide area of our bodies. This somatosensory information reaches the primary somatosensory cortex (S1), and “sensory gating” (suppression of the response for other sensory input) occurs [56]. Additionally, a previous study found that cholinergic neural activity evaluated by the paired-pulse transcranial magnetic stimulation (TMS) paradigm significantly decreased during WI [57]. Cholinergic neural activity enhances the signal-to-noise ratio for sensory input, thereby facilitating the processing of meaningful (associative) inputs and suppressing non-meaningful/irrelevant asynchronous inputs [58,59,60]. Considering these neurophysiological changes during WI, the somatosensory input induced by WI might inhibit the response and processing of HR-related sensory input, which would result in no change in IAcc during WI. However, further studies are needed because the present results could not provide direct evidence of changes in HR-related sensory input. Interestingly, the changes in IAcc scores were significantly correlated with the changes in the not-distracting subscale of the MAIA and the TEA in the present study.

It is possible that attention to sensory input and the ability required for time estimation could be involved in IAcc, although the present study did not show clear evidence thereof. Another possibility could be explained by the suppression of sympathetic nervous activity during WI. Previous studies have reported that WI alters the autonomic nervous system (ANS) response in humans. Several short-duration WI protocols all increased HR variability (HRV), and the greatest impact of WI on HRV is on the high-frequency (HF) component, indicating a shift toward enhanced parasympathetic nervous activity [61,62]. A previous study using transcutaneous auricular vagus nerve stimulation (taVNS), which stimulates the vagus nerve noninvasively and inhibits sympathetic nervous activity, revealed that interoception significantly improved after this stimulation, although cardiac sympathetic nervous activity returned to baseline levels [63]. The authors explained that taNVS could activate the insular cortex, which modulates interoception. It is possible that the temporal inhibition of sympathetic nervous activity during WI could be involved in the improvement of IAcc after WI, although there is no clear result that WI modulates the activity in the insula. IAcc has been reported to improve with feedback information concerning HR. Ring et al. [64] measured IAcc before and after HR feedback and showed that IAcc could be better with HR feedback than without HR feedback, similar to several previous studies [42,65,66]. Additionally, Iodice et al. [67] found that wrong HR feedback caused participants to misinterpret their physiological condition, including interoception. These results confirm that HR feedback strongly affects IAcc. In the present study, participants did not receive HR feedback before and between trials, although similar tasks were repeated three times throughout the experiment. Therefore, IAcc improvement at post WI could not be due to HR feedback.

The present results contradicted our hypothesis that swimmers with continued long-term training in daily life have a specific response to IAcc during WI. This can be attributed to the experimental setting. Swimmers were trained in the supine position at a lower water temperature (26 °C–28 °C) than in the present study (34 °C–35 °C). Considering that water temperature induces physiological and psychological changes [68,69] and adaptation to water temperature differs by age [70] and term [71], the distinct results shown in the present study might depend on water temperature.

In the current study, potential limitations need to be discussed. The first is the cultural aspect. The subjects in this study were Japanese, and the differences of cultural background affect not only somatic awareness [55] and/or preference choice as decision-makers [72], but also interoception [55]. Though the present study cannot provide direct evidence that cultural background affects the interoceptive response for long-term sports training and water immersion, further studies need cross-racial comparisons. Secondly, measuring by the heartbeat counting task or heartbeat detection task is imperfect in the validity of IAcc scores [44,49,51]. Zamariola et al. [51] pointed out the problem of interoceptive accuracy scores massively reflecting under-reports of HR. Knapp-Kline and Kline [44] reported intra-individual detection differences in interoceptive accuracy as measured by the heartbeat detection task. Thus, the results of IAcc scores have not ignored the under-reports of HR and intra-individual differences. These differences are thought to be influenced by time perception [49], knowledge of HR [51], and instruments for HR detection [73]. Indeed, the results showed a negative correlation between the ratio of IAcc and that of TEAcc (Figure 6C). However, the current study was conducted among healthy males; a within-subject design may be useful for the IAcc score study.

## 5. Conclusions

In conclusion, the present results suggest that interoception did not differ in athletes with long-term training, who required multi-modal sensorimotor integration, compared to non-athletes (control participants). Based on the present results, it is possible that sports involving emotional expression (e.g., dance) may be an effective way of increasing interoception through long-term training. On the other hand, because the present results determined that WI significantly increased interoception in all groups, WI may be a useful tool to improve interoception.

## Figures and Tables

**Table 1:** The Multidimensional Assessment of Interoceptive Awareness (MAIA) and the 20-item Toronto Alexithymia Scale (TAS-20).

		Swimmer		Baseball		Control		ANOVA (Environment, Group, and Interaction)	Post Hoc Test
MAIA	Noticing (pre)	3.16	±1.01	3.18	±1.32	2.95	±0.86	F (1.428, 64.26) = 0.435, *p* = 0.649, ηp^2^ = 0.010	
	(WI)	3.13	±0.67	3.23	±0.99	3.03	±0.65	F (2, 45) = 0.162, *p* = 0.851, ηp^2^ = 0.007	
	(post)	3.16	±0.66	3.22	±1.00	3.14	±0.78	F (4, 90) = 0.308, *p* = 0.872, ηp^2^ = 0.014	
	Not-distracting (pre)	2.75	±0.91	2.36	±0.91	2.50	±0.74	F (2, 90) = 0.323, *p* = 0.725, ηp^2^ = 0.007	
	(WI)	2.61	±0.73	2.24	±0.73	2.60	±0.96	F (2, 45) = 1.288, *p* = 0.286, ηp^2^ = 0.054	
	(post)	2.49	±0.82	2.16	±0.58	2.73	±0.88	F (4, 90) = 1.314, *p* = 0.271, ηp^2^ = 0.055	
	Not-worrying (pre)	2.18	±0.69	2.49	±0.80	2.19	±0.60	F (2, 90) = 0.228, *p* = 0.797, ηp^2^ = 0.005	
	(WI)	1.92	±0.60	2.56	±0.75	2.25	±0.59	F (2, 45) = 2.273, *p* = 0.115, ηp^2^ = 0.092	
	(post)	2.16	±0.65	2.53	±0.75	2.15	±0.49	F (4, 90) = 1.702, *p* = 0.159, ηp^2^ = 0.070	
	Attention regulation (pre)	3.02	±0.64	3.12	±0.80	2.58	±0.54	F (2, 90) = 4.0590, *p* = 0.021, ηp^2^ = 0.083	* Pre vs. WI (*p* = 0.025)
	(WI)	2.99	±0.68	3.37	±0.51	2.78	±0.65	F (2, 45) = 3.511, *p* = 0.038, ηp^2^ = 0.135	* Baseball vs. control (*p* = 0.034)
	(post)	2.92	±0.72	3.27	±0.71	2.62	±0.64	F (4, 90) = 1.571, *p* = 0.189, ηp^2^ = 0.065	
	Emotional awareness (pre)	2.98	±0.73	3.57	±0.70	3.26	±0.94	F (2, 90) = 0.055, *p* = 0.946, ηp^2^ = 0.001	
	(WI)	3.11	±0.60	3.41	±0.61	3.35	±0.87	F (2, 45) = 1.804, *p* = 0.176, ηp^2^ = 0.074	
	(post)	2.99	±0.63	3.40	±0.79	3.49	±0.80	F (4, 90) = 1.798, *p* = 0.136, ηp^2^ = 0.074	
	Self-regulation (pre)	3.09	±0.68	3.40	±0.67	2.70	±0.73	F (1.451, 65.286) = 0.220, *p* = 0.730, ηp^2^ = 0.005	
	(WI)	3.10	±0.46	3.57	±0.50	2.67	±0.56	F (2, 45) = 9.738, *p* < 0.001, ηp^2^ = 0.302	* Baseball vs. control (*p* < 0.001)
	(post)	3.15	±0.52	3.48	±0.59	2.69	±0.63	F (4, 90) = 0.293, *p* = 0.882, ηp^2^ = 0.013	
	Body listening (pre)	2.69	±0.86	3.07	±0.68	2.08	±0.86	F (2, 90) = 1.012, *p* = 0.367, ηp^2^ = 0.022	
	(WI)	2.86	±0.69	3.07	±0.84	2.23	±0.96	F (2, 45) = 4.937, *p* = 0.011, ηp^2^ = 0.181	* Baseball vs. control (*p* = 0.010)
	(post)	2.75	±0.89	3.07	±0.76	2.38	±0.93	F (4, 90) = 0.670, *p* = 0.615, ηp^2^ = 0.029	
	Trusting (pre)	3.33	±0.76	3.78	±0.65	3.06	±1.01	F (2, 90) = 1.774, *p* = 0.176, ηp^2^ = 0.038	
	(WI)	3.20	±0.85	3.69	±0.65	2.94	±0.97	F (2, 45) = 3.380, *p* = 0.043, ηp^2^ = 0.131	* Baseball vs. control (*p* = 0.048)
	(post)	3.06	±0.81	3.73	±0.75	3.06	±0.99	F (4, 90) = 0.985, *p* = 0.420, ηp^2^ = 0.042	
TAS-20	Total (pre)	47.24	±8.31	46.93	±7.99	46.69	±8.76	F (2, 90) = 3.354, *p* = 0.039, ηp^2^ = 0.069	
	(WI)	48.35	±7.84	47.40	±8.96	47.50	±9.79	F (2, 45) = 0.170, *p* = 0.844, ηp^2^ = 0.007	
	(post)	50.76	±9.81	47.00	±8.69	48.50	±9.74	F (4, 90) = 1.160, *p* = 0.334, ηp^2^ = 0.049	
	DIF (pre)	15.53	±5.29	13.47	±5.08	13.63	±4.06	F (2, 90) = 3.564, *p* = 0.032, ηp^2^ = 0.073	* Pre vs. post (*p* = 0.049)
	(WI)	16.24	±5.25	13.80	±5.45	14.88	±4.24	F (2, 45) = 1.227, *p* = 0.303, ηp^2^ = 0.052	
	(post)	16.76	±5.45	13.80	±4.71	15.50	±3.86	F (4, 90) = 0.520, *p* = 0.721, ηp^2^ = 0.023	
	DDF (pre)	12.12	±2.21	12.67	±3.79	12.94	±3.53	F (2, 90) = 2.635, *p* = 0.077, ηp^2^ = 0.055	
	(WI)	12.71	±2.11	13.53	±4.58	12.94	±4.22	F (2, 45) = 0.107, *p* = 0.889, ηp^2^ = 0.005	
	(post)	13.18	±2.88	13.20	±4.26	13.56	±4.44	F (4, 90) = 0.549, *p* = 0.700, ηp^2^ = 0.024	
	EOT (pre)	19.59	±2.83	20.80	±4.16	20.13	±4.40	F (1.640, 73.815) = 1.154, *p* = 0.320, ηp^2^ = 0.025	
	(WI)	19.41	±2.62	20.07	±4.23	19.69	±4.39	F (2, 45) = 0.080, *p* = 0.923, ηp^2^ = 0.004	
	(post)	20.82	±3.64	20.00	±4.49	19.44	±4.86	F (4, 90) = 2.570, *p* = 0.043, ηp^2^ = 0.103	

The TAS-20 has three subscales: Difficulty Identifying Feelings (DIF), Difficulty Describing Feelings (DDF), and Externally Oriented Thinking (EOT). * Significantly (*p* < 0.05).

## Supplementary Materials

The following are available online at https://www.mdpi.com/article/10.3390/ijerph181910259/s1, Table S1: Details on participants’ characteristics, Figure S1: IAcc in each type of task, Figure S2: TEA in each type of task.
