# Supplementary material for: Does Long-Term Training in a Water Immersion Environment Change Interoception?
Source: Int J Environ Res Public Health. 2021 Sep 29;18(19):10259. doi: 10.3390/ijerph181910259 (PMC8508048; doi:10.3390/ijerph181910259)
Supplement: Supplementary file 1 [file ijerph-18-10259-s001.zip › ijerph-1364858-supplementary.pdf]

Supplemental material

Interoceptive accuracy

IAcc was evaluated by three types of tasks (25 s, 35 s, and 45 s). Results from the three-factor mixed design ANOVA revealed a significant main effect of "environment" ($F[1.760,3.519] = 9.689$, $p < 0.001$, $\eta p2 = 0.177$), but not other interactions and main effects (Figure S1).

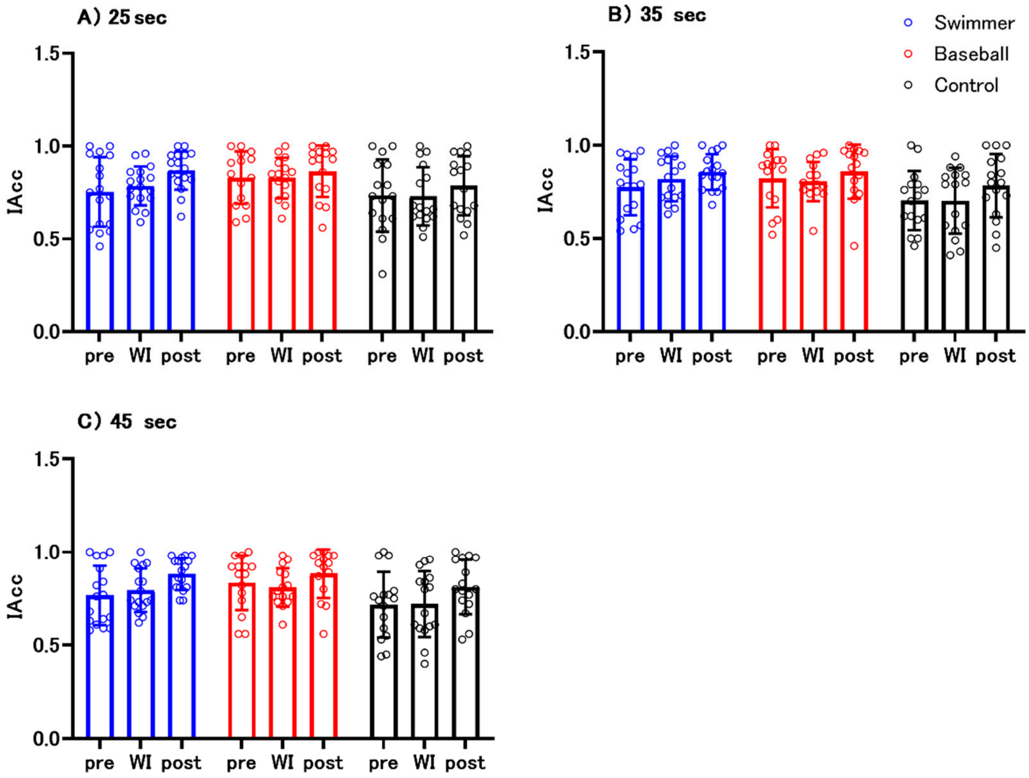

Figure S1. IAcc in each type of task

Time estimation accuracy (TEA)

TEA was evaluated using six types of tasks (25 s, 30 s, 35, s 40 s, 45 s, and 50 s). Results from the three-factor mixed design ANOVA revealed a significant main effect of “environment” ($F[2,90] = 3.699$, $p < 0.029$, $\eta p2 = 0.076$), but not other interactions and main effects (Figure S2).

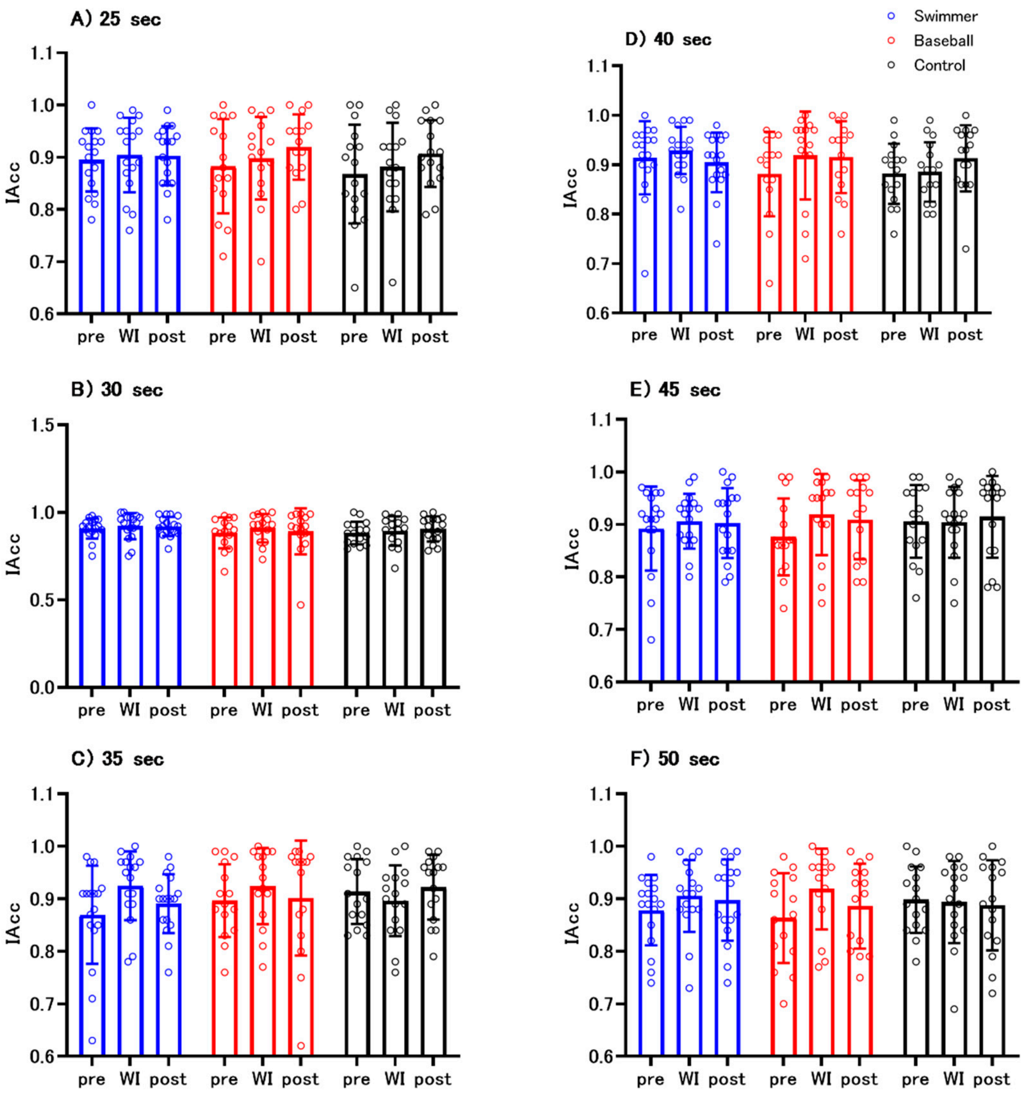

Figure S 2. TEA in each type of task

Table S1. Details on participant's characteristics.

	Sex	Event of swimmers	Height (cm)	Weight (kg)	BMI	Age (y.o.)	Years of training	Performance level (FINA point)
1	M	FR	169	65	23	20	12	723
2	M	FLY	170	75	26	17	17	662
3	M	FLY	163	64	24	10	10	717
4	M	FR	180	72	22	16	16	736
5	M	FR	171	69	24	21	13	737
6	M	BA	177	66	21	18	17	683
7	M	FR	165	60	22	19	15	706
8	M	BR	174	66	22	20	10	844
9	M	FR	167	65	23	18	12	791
10	M	FR	172	66	22	18	11	655
11	M	BA	174	67	22	19	16	674
12	M	FR	175	72	24	18	12	731
13	M	BR	169	74	26	20	16	699
14	M	BR	176	68	22	20	19	788
15	M	FLY	181	75	23	19	10	746
16	M	BR	171	68	23	21	16	832
17	M	BA	166	65	24	20	18	694
AVERAGE			171.7	68.0	23.1	18.5	14.1	730.4
SD			5.1	4.2	1.3	2.6	3.0	55.7

	Sex	Event of Sports	Height (cm)	Weight (kg)	BMI	Age (y.o.)	Years of training
1	M	Baseball	182	84	25	21	11
2	M	Baseball	180	87	27	19	10
3	M	Baseball	178	77	24	21	15
4	M	Baseball	182	78	24	19	10
5	M	Baseball	169	70	25	19	10
6	M	Baseball	178	83	26	19	12
7	M	Baseball	165	68	25	21	12
8	M	Baseball	186	86	25	20	11
9	M	Baseball	180	73	23	22	11
10	M	Baseball	174	71	23	21	9
11	M	Baseball	175	78	25	19	16
12	M	Baseball	170	71	25	19	11
13	M	Baseball	177	81	26	19	9
14	M	Baseball	176	82	26	22	15
15	M	Baseball	173	69	23	19	12
AVERAGE			175.4	75.9	24.6	19.9	11.5
SD			5.5	6.5	1.3	1.2	2.2

	Sex	Event of Sports	Height (cm)	Weight (kg)	BMI	Age (y.o.)	Years of training
1	M	non athletes	168	61	22	19	0
2	M	non athletes	170	68	24	19	0
3	M	non athletes	170	60	21	20	3
4	M	non athletes	170	60	21	19	3
5	M	non athletes	183	86	26	20	0
6	M	non athletes	184	60	18	20	0
7	M	non athletes	166	66	24	19	0
8	M	non athletes	176	51	16	20	0
9	M	non athletes	176	73	24	20	6
10	M	non athletes	168	58	21	20	6
11	M	non athletes	165	52	19	19	0
12	M	non athletes	183	75	22	18	6
13	M	non athletes	163	59	22	20	0
14	M	non athletes	187	70	20	19	0
15	M	non athletes	177	65	21	20	0
16	M	non athletes	163	55	21	20	0
AVERAGE			172.8	63.4	21.2	19.5	2.3
SD			7.9	9.1	2.4	0.6	2.4

FINA point presents swimming performance level. It is normalized to the world record value for each event. FLY, BA, BR, FR, IM are abbreviated words of butterfly stroke, back stroke, breaststroke, free style, individual medley, respectively.
